# Determinants of waterpipe use amongst adolescents in Northern Sweden: a survey of use pattern, risk perception, and environmental factors

**DOI:** 10.1186/s13104-015-1413-4

**Published:** 2015-09-15

**Authors:** Rathi Ramji, Judy Arnetz, Maria Nilsson, Hikmet Jamil, Fredrik Norström, Wasim Maziak, Ywonne Wiklund, Bengt Arnetz

**Affiliations:** Department of Public Health and Caring Sciences, Uppsala University, Uppsala, Sweden; Department of Public Health and Clinical Medicine, Epidemiology and Global Health, Umeå University, Umeå, Sweden; Department of Family Medicine, College of Human Medicine, Michigan State University, 788 Service Road, East Lansing, MI USA; Robert Stempel College of Public Health and Social Work, Florida International University, Miami, FL USA; Västerbotten County Council, Umeå, Sweden

**Keywords:** Hookah, Argile, Depression, Socioecological model

## Abstract

**Background:**

Determinants of waterpipe use in adolescents are believed to differ from those for other tobacco products, but there is a lack of studies of possible social, cultural, or psychological aspects of waterpipe use in this population. This study applied a socioecological model to explore waterpipe use, and its relationship to other tobacco use in Swedish adolescents.

**Methods:**

A total of 106 adolescents who attended an urban high-school in northern Sweden responded to an anonymous questionnaire. Prevalence rates for waterpipe use were examined in relation to socio-demographics, peer pressure, sensation seeking behavior, harm perception, environmental factors, and depression.

**Results:**

Thirty-three percent reported ever having smoked waterpipe (ever use), with 30 % having done so during the last 30 days (current use). Among waterpipe ever users, 60 % had ever smoked cigarettes in comparison to 32 % of non-waterpipe smokers (95 % confidence interval 1.4–7.9). The odds of having ever smoked waterpipe were three times higher among male high school seniors as well as students with lower grades. Waterpipe ever users had three times higher odds of having higher levels of sensation-seeking (95 % confidence interval 1.2–9.5) and scored high on the depression scales (95 % confidence interval 1.6–6.8) than non-users. The odds of waterpipe ever use were four times higher for those who perceived waterpipe products to have pleasant smell compared to cigarettes (95 % confidence interval 1.7–9.8). Waterpipe ever users were twice as likely to have seen waterpipe use on television compared to non-users (95 % confidence interval 1.1–5.7). The odds of having friends who smoked regularly was eight times higher for waterpipe ever users than non-users (95 % confidence interval 2.1–31.2).

**Conclusion:**

The current study reports a high use of waterpipe in a select group of students in northern Sweden. The study adds the importance of looking at socioecological determinants of use, including peer pressure and exposure to media marketing, as well as mental health among users.

## Background

Waterpipe smoking originated in Asia and is widely practiced in the Middle East [1]. The popularity of waterpipe use is partially attributed to a generally held belief that it is less dangerous than cigarette smoking [2, 3]. Similar misbeliefs have persuaded parents towards a more accepting attitude of waterpipe use in adolescents in comparison to smoking cigarettes [4]. However, waterpipe smoke contains high levels of carbon monoxide and other harmful substances that can lead to the same types of morbidity as from smoking cigarettes [5]. Case reports of acute carbon monoxide poisoning from waterpipe smoking have also been documented [6]. Young adults who smoke waterpipe commonly believe that it does not contain tobacco [7]. Although tobacco free waterpipe products are available in the market, some products contain tobacco even if it is not mentioned on the package [7]. Also, toxicity analysis comparing tobacco free waterpipe products with tobacco containing waterpipe products show that smoke from both contained substantial quantities of toxicants [8].

The use of waterpipe among adolescents in the western world has been increasing steadily [9]. According to the World Health Organization (WHO), traditional and new tobacco products are gaining popularity among adolescents as a consequence of exposure to tobacco advertising, promotions and sponsorships [10]. An increased availability of newer forms of tobacco and persistent marketing strategies by tobacco manufacturers have made existing laws promoting adolescent tobacco control less effective [11]. Studies have found an association between greater access to waterpipe products and higher prevalence of use among adolescents [12]. Sixty percent of boys in select regions in Sweden reported to have ever tried waterpipe, compared to 49 % of girls [13]. In another national survey from 2012 on tobacco use in school children, 32 % of the boys and 30 % of girls in grade 9 had ever smoked waterpipe [14]. More than one quarter (27 %) of the Swedish population 15 years and above reported having tried water pipe, a considerably higher average than the 12 % reported for Europe in general [14]. This is in contrast to the fact that regular smoking of cigarettes among Swedish adolescents are among the lowest in Europe [15]. In times of economic hardship, it is also a concern that adolescents consider waterpipe products to be less expensive than cigarettes [16].

Determinants of waterpipe use among adults include contextual (e.g., socioeconomic, cultural and physical environmental conditions), familial, and individual level factors [17, 18]. Adolescents who smoked waterpipes had family members or relatives who smoked waterpipe in their home [19]. In young adults and college students, having friends that smoked waterpipe might influence non-smokers to initiate waterpipe use [2, 20]. It is not known whether the same peer influence is at work in adolescents. Media, in particular social networking, blogging and other internet-based communication, influence the lifestyle of adolescents [21]. Waterpipe videos are reported to be more likely to be watched, liked and commented on by adolescents than cigarette-related videos on YouTube [22]. Having a spare time paid job has been associated with increased use of tobacco and alcohol amongst adolescents [23]. Part-time work might be associated with stress, dealing both with work and school demands, but also increased financial independence from parents, which might facilitate worse lifestyle behavior including waterpipe use. Waterpipe tobacco is available in various flavors, and several studies reported that the smell of waterpipe tobacco is perceived more pleasant than that of other tobacco products, thus promoting waterpipe use in adolescents [3, 19]. Sensation seeking is one important characteristic of an adolescents’ personality [24]. It has been associated with a number of risky behaviors including smoking, heavy drinking, and drug abuse [25].

### Theoretical framework guiding the research analytical strategy

**Fig. 1:**
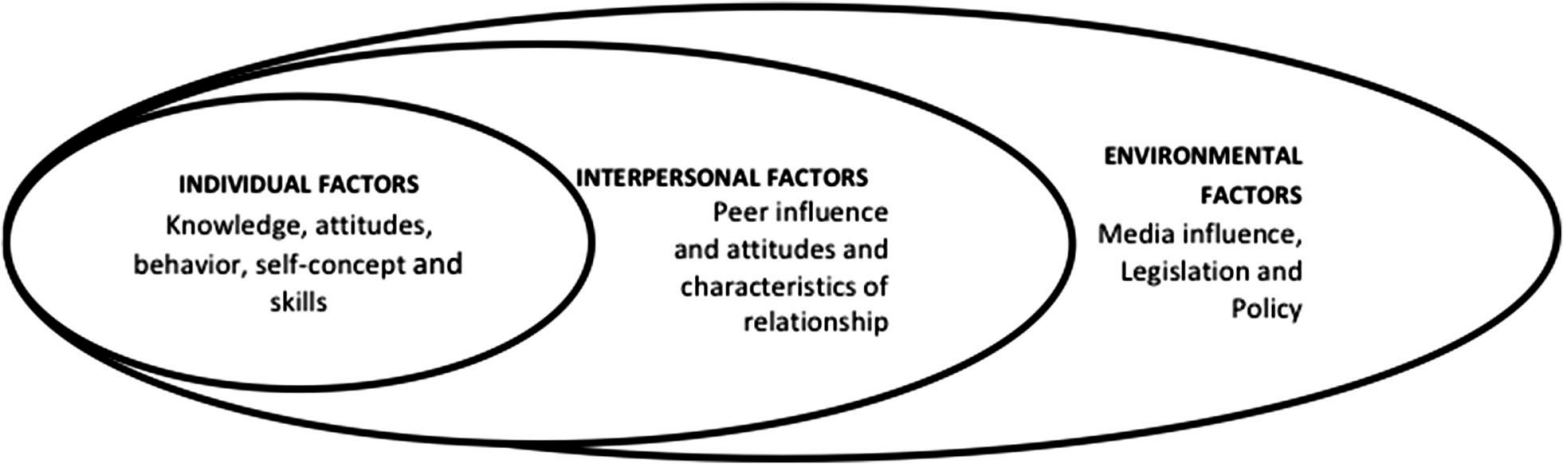
Socioecological model predicting waterpipe smoking based on the Stokol’s theory [26]

The current study uses a socioecological model to examine waterpipe use in adolescents in relation to behavioral health risk and resilience factors. The use of a socioecological model is common in behavioral health studies. However it has not been applied to studies of waterpipe smoking in adolescents. The current study included factors within the modified socioecological model based on Stokol’s theory [26] which is recommended for studying emerging problems in new populations (see Fig. 1). These factors include personal (demographic factors, harm perception, sensation seeking), socio-cultural (ethnicity, religion), peer influence (friends smoking, cultural acceptance), environmental factors (community violence), economic policy mediators (including marketing and product placement), and psychosocial and health factors. They may comprise individual or interdependent elements that might be associated with initiation and prolonged waterpipe use. Identifying these factors and understanding the interconnection between them may enhance our knowledge of waterpipe use in adolescents. This is a critical first step in designing targeted and effective prevention strategies.

### Study aim

The overall aim of this survey was to determine the prevalence of waterpipe use in a sample of adolescents in northern Sweden, and apply a socioecological model to better understand determinants of use. The study also explored factors associated with waterpipe use in comparison to smoking cigarettes. The long-term goal of this research is to develop an evidence-based, socioecological model that will guide the design of intervention trials.

## Methods

### Participants

The study was conducted in January 2013 among a convenience sample of 106 participants, from a large urban high school in the city of Umeå in northern Sweden. Umeå has approximately 120,000 inhabitants and is the largest city north of the capital city of Stockholm. The chosen school is unique in terms of offering many specialized educational programs. Thus students in this school come from various localities in Umeå, and can therefore be considered a representative sample of adolescents in the city. The research team approached the school with the support of the municipality who operated the school, and the school superintendent agreed to have the school participate in the study. The co-ordination between the school and the research team was facilitated by the school’s student counselor. Potential participants were verbally informed about the research goals and volunteered to participate. The teachers also informed all parents about the study via the school website.

### Data collection

The survey was conducted in the classroom during regular school hours. The survey instrument was self-administered. The study was conducted by a research assistant in the absence of any teachers to make sure the students felt comfortable in participating in the survey. All the students who were invited responded to the questionnaire. Student responses were anonymous, with no personal identifier on the survey. The survey was based on a questionnaire used in a prior US waterpipe study [17]. It included questions on demographics, academic performance, sensation seeking behavior, harm perception, depression, peer pressure, personal waterpipe and cigarette smoking habits, ever use of snuff and age of first use of tobacco products. *School performance, that is grades,* was assessed with the question “How is your school performance?” with below average, average or above average as possible choices. Having a paid job was assessed using the question “Do you have a paid job?” (Yes or No). Questions also focused on off-school activities and number of friends. *Self*-*reported stress* and *self*-*rated health* were each accessed using a 10-point scale, ranging from a low of 1 to a high of 10 [27]. *Waterpipe use* included questions regarding ownership of a waterpipe, where and with whom they smoked waterpipe, and reasons for waterpipe use. Ever use was defined as having smoked waterpipe once or more in one’s life time. Current waterpipe use was defined as having smoked waterpipe in the past 30 days. *Beliefs concerning waterpipe* were explored using questions like, “Do you consider waterpipe smoking a form of tobacco use?” (Yes or No). Participants were also asked about their *perceptions of health risks from waterpipe smoking* compared to cigarettes by the following question: “Do you believe that waterpipe smoking is less harmful than cigarette smoking?” (Yes or No). Sensation-seeking was measured using the 8-item Brief Sensation Seeking scale (BSS) [28]. A sample item was “I would love to have new and exciting experiences, even if they are illegal.” Responses were coded on a five point Likert scale [from 1 (Strongly disagree) to 5 (Strongly agree)]. *Depression* was assessed using the Patient Health Questionnaire-9 (Phq-9) [29], including items like, “Little interest or pleasure in doing things” with responses, Not at all, Several days, More than half the days and Nearly every day. *Peer pressure* participants were asked about their peers, for example, “If your friends offer will you smoke waterpipe?”(Yes or No). *Media influence* participants were asked about media placements of waterpipe products using, e.g., Have you ever seen waterpipe advertised on the television?” (Yes or No).

### Statistical analysis

All statistical analyses were performed using IBM SPSS 20 (IBM SPSS Statistics for Windows, Version 20.0. Armonk, NY: IBM Corp). Waterpipe use was analyzed in relation to gender, school grade, school performance, having a paid job, depression, sensation seeking, harm perception, peer pressure, environmental cues for waterpipe use, and ever use of cigarette and ever use of snuff (smokeless tobacco). Chi square tests were performed to identify statistical differences in categorical variables based on waterpipe use. Logistic regression was performed to analyze factors associated with waterpipe ever use. Similar analyses examined risk indicators for waterpipe use in comparison to cigarettes, and to examine the association between waterpipe ever use and media marketing, harm perception, sensation seeking, and peer influence, respectively.

Statistical significance was set at a two-sided *p* value of <0.05.

### Ethical issues

The school principal was informed about the research and provided permission to conduct the study in the school. Our study complied with the Helsinki Declaration. The study did not fall under the Swedish rules requiring approval from the Institution Review Board since it implied minimal risk, did not involve any intervention or clinical trials, and target adolescents aged 16 years and older. The questionnaires and subsequent data files from this study were handled only by the research team after the data collection.

## Results

A total of 106 participants, 68 % (n = 72) girls and 32 % (n = 34) boys, between the ages of 16–19 years, attending grades 10–12 responded to the survey. Ninety-six percent of the participants were born in Sweden, while about 4 % of the participants were born outside Sweden.

Overall, 33 % of respondents reported ever having used waterpipe, with 25 % reporting having smoked waterpipe once or more in the last 30 days. A comparison of sociodemographic characteristics between waterpipe ever users and non-users is presented in Table 1. Waterpipe ever use was significantly higher among boys (44 %) than girls (28 %) and also amongst students in the 11th and 12th grade (37 %) in comparison to students in the 10th grade (20 %). Being depressed and having ever used snuff and cigarettes, were significantly associated with waterpipe ever use. Waterpipe ever use was also significantly higher amongst students who reported worse grades. Waterpipe ever use was not associated with having a paid job. Thirty-five percent reported ever use of both waterpipe and cigarettes, and 25 % reported dual use of waterpipe and snuff. Twenty-two percent of participants had ever tried the combination of cigarette, waterpipe and snuff. Thirty-three percent of the students reported that they had never used waterpipe or any other form of tobacco, including snuff.

**Table 1:** Characteristics of the participants based on waterpipe ever use and non-use

Sociodemographic characteristics	Study participants	Waterpipe ever use	Never smoked waterpipe
	N = 106 N (%)	N = 62 N (%)	N = 44 N (%)
Gender*			
Boys	34 (32)	25 (74)	9 (26)
Girls	72 (68)	37 (51)	35 (49)
Age*			
19–20 years	41 (39)	31 (76)	10 (24)
17–18 years	65 (61)	31 (48)	34 (52)
School grade*			
HS2 and HS3	81 (76)	56 (70)	25 (30)
HS1	25 (24)	6 (24)	19 (76)
School performance*			
Average and below average	65 (61)	43 (66)	22 (34)
Above average	41 (39)	19 (46)	22 (54)
Job			
Yes	25 (24)	15 (60)	10 (40)
No	81 (76)	47 (58)	34 (42)
Depression*			
Yes	28 (26)	21 (75)	7 (25)
No	78 (74)	41 (53)	37 (47)
Cigarette ever use**			
Yes	51 (48)	37 (73)**	14 (27)
No	55 (52)	25 (46)*	30 (54)
Snuff ever use*			
Yes	35 (33)	25 (71)*	10 (29)
No	71 (67)	37 (52)	34 (48)
Sensation seeking score^Ϯ^			
Mean (standard deviation)	3.4 (0.7)	3.6 (0.7)	3.2 (0.7)

* p < 0.05, ** p < 0.001, p values were reported from Chi square test

^Ϯ^ p < 0.05, p values were reported from independent sample t test

### Individual determinants of use

Table 2 reports results from the logistic regression modeling, adjusting for age and gender. Boys had an almost three times higher odds of having ever tried waterpipe in comparison to girls. Odds of performing poorly at school was twice as higher for waterpipe ever users than non-users. Waterpipe ever users had three times higher odds of having ever smoked cigarettes, compared to non-users. Those who reported an ongoing depression had a three times higher odds of being waterpipe ever smokers than non-smokers. Higher levels of sensation seeking were associated with waterpipe ever use than non-use (Table 2).

**Table 2:** Logistic regression examining individual factors associated with Waterpipe ever use (n = 106)

Factors	Crude odds ratio (95 % confidence interval)	Adjusted odds ratio^ϯ^ (95 % confidence interval)
Gender*		
Boys	2.6 (1.1–6.4)	2.9 (1.1–7.3)
Girls (reference)		
Age*		
19–20 years	3.4 (1.4–8.1)	3.6 (1.5–8.8)
17–18 years (reference)		
School grade*		
HS2 and HS3	7.1 (2.5–19.9)	4.7 (1.5–14.6)
HS1 (reference)		
School performance*		
Average and below average	2.3 (1.1–5.1)	2.5 (1.1–5.8)
Above average (reference)		
Job		
Yes	1.1 (0.4–2.7)	1.5 (0.5–4.1)
No (reference)		
Depression*		
Yes	2.7 (1.1–7.1)	3.4 (1.2–9.5)
No (reference)		
Cigarette ever use**		
Yes	3.2 (1.2–7.4)	3.3 (1.4–7.9)
No (reference)		
Snuff ever use*		
Yes	2.3 (1.0–5.5)	2.6 (1.1–7.1)
No (reference)		
Sensation seeking scale score*	2.2 (1.2–4.4)	3.2 (1.6–6.8)

* p < 0.05, ** p < 0.005, were p values for the confidence interval

^ϯ^Adjusted for age and gender

### Socioecological factors

Results of logistic regression examining perceptions of cigarette smoking compared to waterpipe ever use are summarized in Table 3. The odds of waterpipe ever use were three times higher for those who had easier access to waterpipe tobacco than cigarettes. Similarly, waterpipe ever users as compared to non-users perceived waterpipe products to have a more pleasant smell than cigarettes. Results of logistic regression analyses examining waterpipe use in relation to media and marketing factors are summarized in Table 4. Waterpipe ever use had a two times higher odds of having seen waterpipe use on television in comparison to non-users. Also, waterpipe ever users had a three times higher odds of not perceiving waterpipe smoking to be unhealthy when they watched people smoking on television or film, compared to those who have never tried waterpipe smoking. There was no significant association between other marketing strategies including advertising and sales in public and waterpipe ever use.

**Table 3:** Logistic regression examining perceptions of cigarette smoking associated with waterpipe ever use (n = 106)

In comparison to cigarette ever use	Study participants	Waterpipe ever use		Crude odds ratio (95 % confidence interval)	Adjusted odds ratio^ϯ^ (95 % confidence interval)
	N = 106 N (%)	Yes N = 62 N (%)	No N = 44 N (%)		
Harm perception cigarette vs. waterpipe					
Less harmful	82 (77)	51 (82)	31 (71)	1.9 (0.7–4.8)	1.6 (0.6–4.3)
More harmful (reference)	24 (23)	11 (18)	13 (29)		
sEasy to get-away with cigarette vs. waterpipe?					
Easy to get-away	43 (59)	29 (71)	14 (52)	2.2 (1.0–5.1)	2.1 (0.9–5.0)
Difficult to get way (reference)	63 (41)	33 (29)	30 (48)		
Parental approval cigarette vs. waterpipe?					
More likely approval	84 (79)	44 (71)	38 (86)	0.4 (0.1–1.1)	0.4 (0.1–1.1)
Approval will be about same (reference)	24 (21)	18 (29)	6 (14)		
Cost cigarette vs. waterpipe?					
Less expensive	10 (9)	7 (11)	3 (7)	1.7 (0.4–7.1)	3.1 (0.7–14.0)
More expensive (reference)	96 (91)	55 (89)	41 (93)		
Accessibility cigarette vs. waterpipe?*					
Easier access	28 (26)	21 (34)	7 (16)	2.7 (1.1–7.1)	3.2 (1.1–8.9)
Difficult to access (reference)	78 (74)	41 (66)	37 (84)		
Smell cigarette vs. waterpipe?*					
Much better	62 (58)	46 (74)	16 (36)	5.1 (2.2–11.6)	4.1 (1.7–9.8)
About the same (reference)	44 (42)	16 (26)	28 (64)		

* p < 0.05, p values for the confidence interval

^ϯ^Adjusted for age and gender

**Table 4:** Logistic regression examining media and marketing factors associated with Waterpipe ever use (n = 106)

Media marketing factors	Study participants	Waterpipe ever use		Crude odds ratio (95 % confidence interval)	Adjusted odds ratio^ϯ^ (95 % confidence interval)
	N = 106 N (%)	Yes N = 62 N (%)	No N = 44 N (%)		
Waterpipe use in television/films*					
Seen	66 (62)	44 (71)	22 (50)	2.4 (1.1–5.5)	2.4 (1.1–5.7)
Not seen (reference)	40 (38)	18 (29)	22 (50)		
Advertisements on waterpipe products					
Seen	91 (86)	55 (89)	36 (82)	1.7 (0.6–5.2)	1.4 (0.4–4.7)
Not seen (reference)	15 (14)	7 (11)	8 (18)		
Waterpipe products for sale in public					
Seen	60 (57)	39 (63)	21 (48)	1.8 (0.8–4.0)	1.8 (0.8–4.1)
Not seen (reference)	46 (43)	23 (37)	23 (52)		
Waterpipe use in tv/films perceived unhealthy*					
No	31 (29)	38 (61)	7 (16)	3.3 (1.3–8.7)	3.2 (1.2–8.9)
Yes (reference)	75 (71)	24 (39)	37 (84)		
Waterpipe use in tv/films perceived cool					
Yes	85 (80)	49 (79)	36 (82)	0.8 (0.3–2.2)	0.9 (0.3–2.7)
No (reference)	21 (20)	13 (21)	8 (18)		

* p < 0.05, were p values for the confidence interval

^ϯ^Adjusted for age and gender

There was no significant association between perceptions of harms associated with waterpipe ever use. However, waterpipe ever users had two times a higher odd of believing that waterpipe smoking makes users cool and fit (Table 5).

**Table 5:** Logistic regression examining perceptions of harm associated with Waterpipe ever use (n = 106)

Harm perception	Study participants	Waterpipe ever use		Crude odds ratio (95 % confidence interval)	Adjusted odds ratio^ϯ^ (95 % confidence interval)
	N = 106 N (%)	Yes N = 62 N (%)	No N = 44 N (%)		
Do you consider waterpipe smoking as a form of tobacco use?					
No	49 (46)	29 (47)	20 (45)	1.5 (0.5–2.3)	1.2 (0.5–2.7)
Yes (reference)	57 (54)	33 (53)	24 (55)		
Is second hand waterpipe smoking harmful?					
No	66 (62)	39 (63)	27 (61)	1.1 (0.5–2.4)	1.3 (0.5–2.9)
Yes (reference)	40 (38)	23 (37)	17 (39)		
There is no risk at all smoking waterpipe for the first few years					
Yes	42 (40)	26 (42)	16 (36)	1.3 (0.5–2.8)	1.1 (0.1–2.4)
No (reference)	64 (60)	36 (58)	28 (64)		
Every puff of waterpipe smoke causes a bit of harm					
No	68 (64)	41 (66)	27 (61)	1.2 (0.6–2.7)	1.2 (0.5–2.8)
Yes (reference)	38 (36)	21 (34)	17 (39)		
Smoking waterpipe for an hour daily is harmful					
No	37 (35)	22 (36)	15 (34)	1.1 (0.5–2.4)	0.9 (0.4–2.2)
Yes (reference)	69 (65)	40 (64)	29 (66)		
Waterpipe smoking makes users cool and fit*					
Yes	44 (42)	31 (50)	15 (34)	1.9 (0.9–4.3)	2.6 (1.1–6.4)
No (reference)	62 (58)	31 (50)	29 (66)		

* p < 0.05, were p values for the confidence interval

^ϯ^Adjusted for age and gender

Waterpipe ever users also had higher odds of having friends who smoked regularly than those who did not smoke waterpipe. Waterpipe ever users had nearly three times higher odds of accepting an offer to smoke waterpipe from smoking friends than non-waterpipe smokers (Table 6).

**Table 6:** Logistic regression examining peer pressure factors associated with Waterpipe ever use (n = 106)

Peer pressure	Study participants	Waterpipe ever use		Crude odds ratio (95 % confidence interval)	Adjusted odds ratio^ϯ^ (95 % confidence interval)
	N = 106 N (%)	Yes N = 62 N (%)	No N = 44 N (%)		
Have friends who smoke waterpipe regularly*					
Yes	21 (20)	19 (31)	3 (7)	6.1 (1.7–22.0)	8.3 (2.1–31.2)
No (reference)	84 (80)	43 (69)	41 (93)		
Waterpipe users have more friends					
Yes	43 (41)	28 (45)	15 (34)	1.6 (0.7–3.5)	2.1 (0.8–4.8)
No (reference)	63 (59)	34 (55)	29 (66)		
If your friends invite will you smoke waterpipe?*					
Yes	61 (57)	42 (68)	19 (43)	2.8 (1.2–6.4)	2.8 (1.2–6.7)
No (reference)	45 (43)	20 (32)	25 (57)		

* p < 0.05, were p values for the confidence interval

^ϯ^Adjusted for age and gender

### Non-smokers

Sixty-one percent of those who did not smoke waterpipe perceived second hand smoking as not harmful in comparison to 56 % of waterpipe ever users. Thirty-five percent of the entire study population believed smoking waterpipe 1 h daily was not harmful, with no significant differences across groups (Table 5). Forty-three percent of those who never smoked waterpipe versus 68 % of waterpipe ever users reported that they would smoke if their friends’ invited them to do so (Table 6).

## Discussion

Thirty-three percent of the participants had tested waterpipe smoking and 25 % were current waterpipe users, which is in line with prior work in adolescents [12, 14, 30]. Our findings support prior national studies that report waterpipe ever use to be in the 50 % range in high school students in Sweden [31]. However, we add to current knowledge of the prevalence of use of waterpipe in adolescents, by exploring the relevance of a socioecological model towards identifying determinants of use.

Boys had more often tried waterpipe smoking than girls, which is in line with the other studies [31]. The present study also highlights the strong association between waterpipe use and consumption of other tobacco products, foremost cigarettes and snuff, in adolescents. Our results on the dual use of waterpipe and cigarette in adolescents are in concurrence with previous studies in young people [32–34]. Our novel data on the high co-use of waterpipe and snuff in the current study raises concerns as to differential tobacco consumption entry gateways in adolescents. In order to further elucidate the temporal relationships between different gateways into the use of various tobacco products, prospective studies are needed. A Danish study of students in grades 8–10 reported that waterpipe use at baseline predicted increased risk of becoming a regular cigarette smoker at the follow-up, especially for boys. Moreover, there may be a dose–response relationship between intensity of waterpipe use and the risk for becoming a regular cigarette smoker [35]. Since most of the existing studies of adolescents, including our own, are cross-sectional and there are only regional, cross-sectional panel reports from Sweden [14, 36], it is not possible to confirm whether waterpipe serves as a gateway to cigarettes or other tobacco products. However, as discussed above, the high concurrent rates between waterpipe use and other means to consume tobacco products raise a concern as to the risk that waterpipe use promotes nicotine dependency in adolescents, or promotes the use of other tobacco products.

In concurrence with previous studies on middle and high school students, waterpipe ever users in comparison to those who never smoked waterpipe believed that waterpipe use makes them cool and fit compared to cigarettes [37]. Concerning perceptions of harm, a large proportion of both waterpipe ever users and non-users believed that second hand waterpipe smoke was not dangerous. Furthermore, they also assumed that smoking waterpipe 1 h a day was not dangerous. Both waterpipe ever users and non-users reported waterpipe smoking to be less harmful than cigarettes, in line with findings from other studies of college students [38]. Nearly 80 % of waterpipe ever users believe that waterpipe smoking is less harmful than cigarettes in comparison to 70 % in non-waterpipe smokers. This suggests a risk that non-waterpipe smokers may more readily try waterpipe smoking, as opposed to cigarette smoking in the future. Our study also shows that the sweet smell from waterpipe smoke encourages adolescents into smoking waterpipe compared to cigarettes. The increasing demand for waterpipe products has been partially attributed to its pleasant smell, many related to fruit [39]. It is a misconception that waterpipe smoking is less dangerous for one’s health than cigarette smoking [1]. To the contrary, recent research reports that waterpipe smoking is as dangerous, or even worse, than cigarette smoking, and has been associated with numerous serious health outcomes, including lung cancer, leukemia, respiratory illness including lung dysfunction, low birth-weight and periodontal disease [6, 7, 40, 41]. We found that an overwhelming majority of the respondents including both waterpipe smokers and non-smokers perceived smoking waterpipe an hour a day, or for a few years and second hand smoke as not at all harmful.

Waterpipe ever users more often had friends who smoked waterpipe which is in agreement with the Danish studies on high school students [35] and studies from secondary school students in London [33]. Interestingly, 40 % of non-waterpipe smokers reported that they would try waterpipe smoking if their friends invited them. This indicates that peer influence might promote initiation of waterpipe smoking in adolescents. Waterpipe smokers reported higher levels of sensation seeking than non-smokers. According to previous studies in primary school children, sensation seeking behavior was associated with waterpipe use [42]. These finding highlights the need to put waterpipe use into a broader conceptual model of relevance for adolescents and their sensation and novelty seeking behaviors [42]. Compared to non-users, waterpipe ever users had not only noticed waterpipe use in television and films more often but had also less often perceived it as unhealthy when they watched people smoking waterpipes on TV shows or films. It may be that waterpipe ever smokers enjoy watching others smoking and themselves get motivated to smoke more. The questions on media marketing that were part of our survey have not been used before in terms of waterpipe smoking. However, there are related studies on media and youth cigarette smoking behavior, showing that advertising and cigarette smoking on movies had an impact cigarette smoking in adolescents [43].

### Limitations

Our study only included teenagers from one school in Umeå city. Although this is a cosmopolitan city, it is not necessarily representative for Sweden as a whole. However, our results are similar to those reported from other regional studies in Sweden [44]. Participation in this study was voluntary and the results may have been affected by selection bias. However, the majority of school children participated in the survey and we therefore do not believe there was any systematic response bias. Our survey was based on self-reports, and results may have been affected by single-method bias and recall bias. For example, self-reported school performance could have been a source of information bias. However, similar measures of school performance were utilized previously by other researchers studying grades and cigarette smoking [45–47], and the pattern of relationships between school performance and waterpipe use was as hypothesized, lending support to the validity of the measure. There may also be a risk of underreporting other tobacco use among the students, foremost cigarettes, due to the decreased social acceptance of such tobacco products and age restrictions for purchase. Previous studies on the validity of self-reported tobacco use among Swedish adolescents have suggested that young people in Sweden are independent and their answers concerning tobacco are unbiased [47].

## Conclusions

Results point to a high prevalence of waterpipe use, and an association between having ever smoked cigarettes as well as snuff, and waterpipe ever use. The study also highlights the need to apply a socioecological model to further our understanding of determinants of use in adolescents. Such knowledge is critical in being able to design effective preventive strategies. Our results also highlight the importance of including waterpipe use in tobacco prevention education as well as tobacco control regulations aimed at adolescents, an age where life-time dependency on nicotine is most likely to be established. The prevalent use of waterpipe also indicates a need to inform the students, school staff and parents about the health risks from waterpipe smoking.
